# Correlation Between Stress and Quality of Life Experienced by Caregivers: Perception of a Group of Healthcare Professionals

**DOI:** 10.2174/1874434601711010135

**Published:** 2017-09-27

**Authors:** Bhárbara Karolline Rodrigues Silva, Fernando Rodrigues Peixoto Quaresma, Erika da Silva Maciel, Francisco Winter dos Santos Figueiredo, Jonathan Souza Sarraf, Fernando Adami

**Affiliations:** 1 Epidemiology and Data Analysis Laboratory, Faculdade de Medicina do ABC (FMABC) - Santo André-SP, Brazil.; 2 Faculdade de Medicina do ABC (FMABC), Lutheran University Center of Palmas -TO and Federal University of the State of Tocantins (UFT) - Palmas-TO, Brazil.; 3 Department of Medical Sciences, at the University of São Paulo (USP), Federal University of the Tocantins (UFT) - Miracema-TO, Brazil.; 4 Faculdade de Medicina do ABC (FMABC), Epidemiology and Data Analysis Laboratory, Faculdade de Medicina do ABC (FMABC) - Santo André-SP, Brazil.; 5 Federal University of the State of Pará (UFPA) - Belém-PA, Brazil.; 6 Faculty of Public Health at the University of São Paulo (USP), Faculdade de Medicina do ABC (FMABC), Laboratory of Epidemiology and Data Analysis, Faculdade de Medicina do ABC (FMABC) - Santo André-SP, Brazil.

**Keywords:** Quality of life, Stress, Healthcare professional, Self-perception, Prompt service units

## Abstract

**Aim:**

This study aims to evaluate the relationship between perceived level of stress and quality of life of professionals working in Prompt Service Units in the city of Palmas, Tocantins.

**Methods:**

A cross-sectional study was performed among 164 professionals from Prompt Service Units. Stress levels were evaluated using the Perceived Stress Scale. The WHOQOL-bref was used to evaluate the perception of quality of life. Quantitative variables distribution was evaluated using Shapiro-Wilk's test. For the analyses of correlations among perceived level of stress, total quality of life score, and the physical health domain of the WHOQOL-bref, Pearson's correlation test was applied. The significance level adopted for this trial was 95%. The study was approved by the Committee of Ethics in Research with Human Beings.

**Results:**

When assessing the perceived level of stress with the total quality of life score, there was no significant correlation between those variables. However, there was an association between the perceived level of stress and the physical health domain of quality of life.

**Conclusion:**

Perceived quality of life was correlated with the physical health domain, and this result reinforces the importance of the facets that make up this area.

## 1 INTRODUCTION

Stress among healthcare professionals has been observed as an occupational risk factor in Brazilian and American professional sectors [1].

Faced with stressors, an individual experiences increasing stress. This process may become a risk if the body does not reestablish homeostasis as a mechanism to adapt to such stressors [2]. Emotional exhaustion, general fatigue, and physical waste are consequences that may lead to Burnout Syndrome or Occupational Burnout Syndrome [3, 4].

Prompt Service Units (PSU) are health centers designed to deal with diseases of intermediate complexity, hierarchically allocated in between the Basic Health Unit and the Hospital Network. They are characterized by a closed environment and keeping close contact with human suffering, pain, and death, its constant attention leading, inevitably, to quality of life repercussions [5, 6].

On health evaluations, the identification of perceived stress has become an important variable considered for occupational health diagnosis [7, 8] and presents itself as a point for feasibly elaborating more efficient interventional strategies [9].

Besides, health professions occupy a prominent place when evaluating stress regarding labor dynamics [10, 11]. There are negative repercussions for employees’ health and for the quality of assistance provided [12].

In this context, one must consider the fact that the lifestyle has become renowned as one of the most important determinants of health and quality of life of individuals and communities [13], so the relevance of this work is clear as stressors in the work of these professionals are the main precursor of a negative perception of one's quality of life.

Thus, the present article aims to discuss the relationship between the perceived amount of stress and the quality of life of PSU working professionals in the city of Palmas, Tocantins.

## 2 MATERIALS AND METHODS

A cross-sectional study performed with a team of 164 multidisciplinary participants present at the PSU of the city of Palmas, Tocantins' capital, was approved by the Committee of Ethics in Research with Human Beings, under the identification of do CAAE 07564412.0.00005516.

The initial sample included all the professionals of the Palmas Pronto Care Units, approximately 250 professionals. However, among the exclusion criteria were those who were on vacation, who did not respond correctly to the questionnaires, who refused to sign the Free and Informed Consent Term, and/or who were not found within the data collection period stipulated by the researchers. A total of 164 professionals participated in the study.

The city has 39 Primary Healthcare Units and 2 PSUs, one of which is located in the northern region and the other in the southern region. All participants were multidisciplinary professionals with Higher Educational Level (Physician, Nurse, Dentist, Social Assistant, and Pharmacist), Technical Level (Nursing Technician, Dental Assistant, Laboratory Technician, and X-Ray Technician), and Medium Level (Security Guard, Administrative Assistant, General Services Assistant, and Stretcher Bearer). Professionals who answered less than 20% of the questions asked or who refused to take part in the trial were excluded from the research.

The professionals were invited to participate in the study, which took the form of an interview, in the workplace after explaining the objective and asking them to sign the Free and Informed Consent Form.

Sociodemographic variables evaluated were sex, profession, PSU, employment relationship, working hours, service time, number of workplaces, and income.

The Perceived Stress Scale - The Stress Perception Scale has demonstrated reliability and evidence of validity regarding actions of risk and health perception. It was proposed to evaluate stress from thoughts and feelings related to the events occurring in the last 30 days as a global measure. Composed of 10 multiple choice questions, it was to evaluate individual general stress [14].

To evaluate perceived quality of life, an instrument validated by the World Health Organization (WHO), the WHOQOL-bref, was used [15, 16]. It is composed of 26 items, 2 questions of which are broad and the other 24 are divided into 4 different domains: 1-physical health, 2-psychological health, 3-social relationships, and 4-environment [17]. The results were stratified for each domain with the understanding that the original division is a characterization of various aspects of one's quality of life.

Qualitative variables were described by absolute and relative frequency. The distribution of quantitative variables was evaluated using the Shapiro-Wilk test. For the domains that did not present normal distribution, score cubic transformation was used; quadratic transformation was used for total quality of life score.

Aiming to analyze correlation between the perceived level of stress, total quality of life score, and the physical health domain of the WHOQOL-bref, Pearson's correlation test was applied. The significance level adopted for this trial was 95%. The statistical software utilized was Stata 11.0.

## 3 RESULTS

In this study, 164 participants took part in the trial. The mean age was 38.13 years (SD = 9.97). Sociodemographic characteristics, profession, PSU in which the participant works, employee relationship, service time, number of places worked, income, and professional level are available in (Table **1**).

Regarding perceived quality of life (Table **2**), the Psychological domain obtained a higher score 14.4 (SD = 1.9). Regarding perceived stress, the average score was 16.7 (SD = 6.7).

**Fig. (1):**
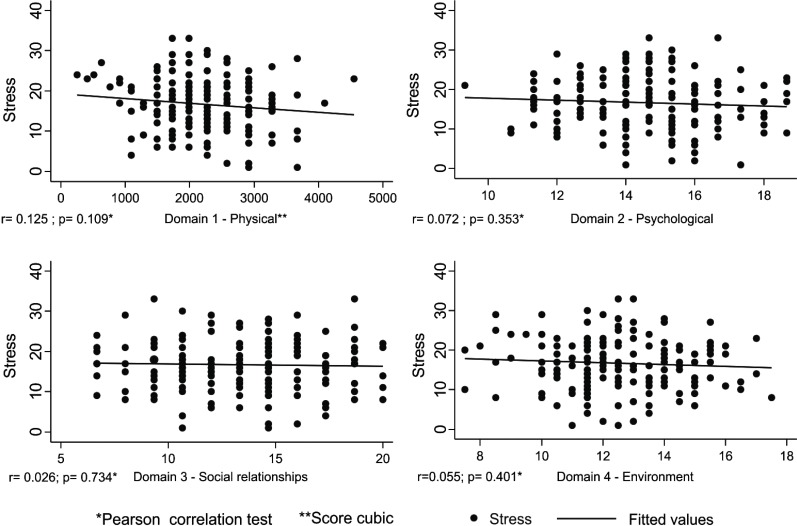
Correlation between the level of perceived stress and the domains of quality of life (cubed).

According to the WHOQOL-bref, no association could be identified between the level of perceived stress and the domains of quality of life (Fig. **1**).

## 4 DISCUSSION

When assessing the relationship between the perceived level of stress and the quality of life of professionals working in Emergency Care Units, no significant relationship was found.

To understand the observed relationship in our trial, it is crucial to consider that the WHOQOL-bref physical health domain is made up by facets that have some type of relationship with basic human needs—for instance, physical pain and discomfort, energy and fatigue, sleep and rest, daily life activities, dependence on medications or treatment, and work capacity [18].

It is a fact that painful and/or uncomfortable situations may negatively reflect on one's quality of life, increasing stress levels. Besides, nursing professionals (majority in this trial n = 96) often experience back pain and musculoskeletal injuries, often due to the posture adopted during the work activity [1].

Regarding energy and fatigue, it has been observed that most of the stressors of healthcare professionals originate with characteristics of the work environment, especially overcrowding, poor working conditions, lack of materials, and work overload [18, 19].

These circumstances, along with working in shifts, which leads to a decline in performance [20], and the practice of accumulating more than one job [21] to increase the income, create overwork, tiredness, lack of adequate rest, musculoskeletal pain [22], and, inevitably, decreased energy and increased fatigue as a consequence of stress.

During a considerable part of his or her life, the employee who works in shifts goes in the opposite direction of the “daytime society,” not only during night shifts but also in the daytime and on weekends and holidays [20]. This affects one's energy level, working capacity, and daily life activities [21].

Furthermore, impairment of the quality of life of healthcare professionals can influence the quality of service delivery, bringing losses across organizational dynamics, including to the patients [18].

In an integrative review study, it was observed that physical exercise, massage therapy, music, and relaxation have been used as coping strategies against labor-related stressors in healthcare professionals [1].

Aid and social support have also been associated with reduced levels of stress in health professionals, especially the support of supervisors [19]. Another alternative that has been reported is the possibility of reducing working time for healthcare professionals [23], one action that, in Brazil, can encourage adherence to more than a working relationship and have a reverse effect on the indicators assessed.

Among the interventions, emphasis is being given to physical exercise programs because they contribute strategies for improving quality of life and mental health and reducing stress and anxiety in the short term and for reducing depression and mood alternations and improving self-esteem in the long term [24].

In a review study that evaluated studies whose aim was to draw a relationship between physical activity and a positive perception of quality of life, it was observed that there is a positive association between these variables and that this relationship is independent of the domains of Quality of Life analyzed [25].

Regular physical activity appears to be related to lower stress levels in workers [26, 27] and healthcare professionals working in the emergency care network [6].

Thus, since the physical health domain has been the most affected by healthcare professionals’ working conditions [22], it is understood that an alternative would be to encourage the practice of regular physical activity to improve perceived quality of life.

However, it is important to highlight some limitations related to this study, regarding sample selection, data related to the practice of physical activity, amount of absences from work by stress, factors such as salary income of professionals can significantly alter the quality of life of these professionals, while physicians claim to have a higher total income than nurses, taking into account Since most of the time the workload of nurses is greater than that of physicians, has more employment ties and is more susceptible to emotional discharges, due to the fact that they receive less than other professionals because of the work done, consequently, altering the quality of life [18, 28].

## CONCLUSION

Results indicate an association between physical health and the stress perception regarding healthcare professionals. As the scores of the physical domain facets diminish, the perceived stress levels increase, which leads to the conclusion that investing in actions considering the items that make up the facets of the physical domain can favor the promotion of health professionals; in this context, regular physical activity can be an interesting measure.

It is important to emphasize the need for continuity of studies about quality of life in this population. Moreover, interventions to improve quality of life prevent future expenses with professionals removed from work because of disease and, consequently, improve patient care. Therefore, the quality of life at work corresponds to the values of a more humane and healthy life.

## Figures and Tables

**Table 1:** Characterization regarding sex, PSU unity of work, employee relationship, service time, number of places worked, income and educational level.

Characteristics	n	%
**Gender**		
Male	58	35.3
Female	106	64.6
**Level of schooling**		
Higher level	55	33.53
Technical level	90	54,88
Middle level	19	11,59
**Profession**		
Nurse	35	21.34
Technical Nurse	61	37.19
Physician	10	6.09
Dentist	4	2.43
Dental Assistant	4	2.43
Social Assistant	4	2.43
Laboratory Technician	1	0.60
X-Ray Technician	4	2.43
Pharmacist	2	1.21
Guard	5	3.04
Administrative Assistant	20	12.19
General Services Assistant	10	6.09
Stretcher bearer	4	2.43
**Unidade UFPA**		
PSU North	107	65.20
PSU South	57	34.80
Employment relationship		
Gazetted	139	84.80
Hired	25	15.20
**Service Time**		
< 1 year	67	40.85
1 to 5 years	23	14.02
6 to 10 years	26	15.85
11 to 19 years	48	29.26
Number of Workplaces		
One	72	43.9
Two	75	45.7
Three	17	10.4
**Income**		
Not discriminated	20	12.19
790,00 to 5.000,00	98	59.75
6.000,00 to 9.000,00	30	18.29
10.000,00 to 19.000,00	11	6.70
>= 20.000,00	5	3.04

PSU= Prompt Service Unit.

**Table 2:** Evaluation of stress, quality of life by domain and general score of quality of life.

	Mean (SD)	Max ; Min
Stress	16.7 (6.7)	1.0 ; 33.0
**Domain**		
Physical	12.8 (1.6)	16.6 ; 6.3
Psicological	14.4 (1.9)	18.7 ; 9.3
Social Relationship	13.5 (3.3)	20.00 ; 6.7
Environment	12.6 (2.0)	17.5 ; 7.5
General score of quality of life	9.6 (3.8)	20.0 ; 4.0
